# Emotional Intolerance and Core Features of Anorexia Nervosa: A Dynamic Interaction during Inpatient Treatment? Results from a Longitudinal Diary Study

**DOI:** 10.1371/journal.pone.0154701

**Published:** 2016-05-18

**Authors:** Esther Stroe-Kunold, Hans-Christoph Friederich, Tatjana Stadnitski, Daniela Wesche, Wolfgang Herzog, Michael Schwab, Beate Wild

**Affiliations:** 1 Department of General Internal Medicine and Psychosomatics, Medical University Hospital Heidelberg, Heidelberg, Germany; 2 Department of Psychological Methods and Statistics, University Ulm, Ulm, Germany; 3 Department of Psychosomatic Medicine and Psychotherapy, LVR-Clinics, University Düsseldorf, Düsseldorf, Germany; University Hospital of Bellvitge-IDIBELL; CIBER Fisiopatología Obesidad y Nutrición (CIBERObn), Instituto Salud Carlos III; Department of Clinical Sciences, School of Medicine, University of Barcelona, Spain, SPAIN

## Abstract

**Objective:**

The role of emotion dysregulation with regard to the psychopathology of anorexia nervosa (AN) is increasingly discussed. It is both assumed that AN symptoms have an impact on difficulties in tolerating aversive emotions and that—conversely—emotion dysregulation influences AN. To date, such conclusions are drawn on the basis of cross-sectional data not allowing for inferences on the temporal dynamics. The current study investigates the longitudinal interaction between emotional intolerance and core AN symptoms over the course of inpatient treatment by comparing patients with high (BMI<15 kg/m^2^) vs. low symptom severity (HSS vs. LSS).

**Method:**

The study adopted a longitudinal, process-oriented design with N = 16 analysed electronic diaries. Throughout the course of their inpatient treatment, the patients answered questions daily about emotional intolerance and their AN-specific cognitions and behaviours. The temporal dynamics between emotional intolerance and these variables were analysed using a multivariate time series approach.

**Results:**

The time series of the processes under investigation adequately reflected the individual treatment courses. The majority of significant linear time trends was found for HSS patients. Most importantly, analysis revealed significant temporal interactions between emotional intolerance and AN symptoms in almost 70% of HSS patients. Thereby, up to 37% of variance in eating restraint and up to 23% in weight concern could be attributed to changes in emotional intolerance.

**Conclusions:**

The findings support the notion that intolerable unpleasant emotions in severely affected AN patients influence their psychopathology. Additionally, time series analysis outlined the inter-individual heterogeneity of psychosomatic treatment courses of AN patients.

## Introduction

Anorexia nervosa (AN) is a serious eating disorder with the highest mortality rate among all mental disorders [1;2]. It is characterised by a chronic course with frequent relapse [3;4]. Accompanied by an intense fear of weight gain and a disturbance of body image, the essential characteristic of AN is one’s inability to maintain a minimum normal weight for age and height.

Unfortunately, high treatment resistance to available interventions poses a crucial challenge to psychotherapists working with AN patients [5;6]. Within the framework of their cognitive-interpersonal maintenance model, Schmidt and Treasure (2006) suggested emotional avoidance or intolerance as one maintenance factor of anorexia nervosa [7]. In accordance with this, AN is described as a disorder of emotion dysregulation in recent research (e.g., [8]). As another example, Kyriacou, Easter & Tchanturia (2009) showed that patients with AN describe a form of emotional numbing as a result of the disorder [9]. Other studies have hypothesised that anorexic symptoms serve as a dysfunctional behaviour to regulate aversive emotions (e.g., [10–13]).

At the same time, one can conclude that the inability to tolerate intense emotions hinders patients from experiencing positive social situations, thereby deepening disorder-related behaviours and interfering with a positive interpersonal learning process. To this end, Brockmeyer et al. (2013) investigated the relation between the alleviation of aversive emotional responses and anorexic symptoms and found that the lower the body weight of AN patients, the fewer negative emotions were retrieved in sad autobiographical memories [14]. In a previous longitudinal single-case study, we hypothesised that if—as assumed by Schmidt and Treasure (2006)–emotional avoidance maintains the disorder, then changes in this variable should correspond to changes in other relevant psychosocial aspects. The results suggested that, for this single patient, emotional intolerance played a central role in the interacting system of various maintaining psychosocial variables [15]. This finding is yet to be confirmed by a study investigating a larger number of AN patients. Also, AN-specific symptom variables should be included in the model for a better understanding of psychosomatic patterns of AN.

Summarising the findings on emotional regulation in AN patients in recent literature, it is both assumed that AN symptoms have an impact on the inability to tolerate aversive emotions and that—conversely—emotion dysregulation influences or even maintains AN psychopathology.

The role of emotion regulation in AN patients needs to be reflected against the broader background of aetiological models of the disorder that are currently discussed. As one example, the model recently outlined by O’Hara, Campbell and Schmidt (2015) suggested AN to be a reward-based learned behaviour in which distorted cognitions related to eating, weight, and shape alter functioning of the striatal reward system [16]. In contrast to this, Södersten, Bergh, Leon, and Zandian (2016) discussed the aetiology of AN against the background of the altered dopamine status of AN patients suggesting that a brain abnormality underlies their complex emotional disorder [17]. Hereby, the authors concluded that the emotional symptoms of AN patients appear to be a consequence of starvation. Another aetiological approach was outlined by Riva (2014) who suggested that AN is the outcome of a disturbed body experience [18]. He concluded that AN patients may be impaired in their ability of updating a negative body representation stored in autobiographical memory by contrasting real-time sensorimotor and proprioceptive data. Regarding the maintenance model of AN previously suggested by Schmidt and Treasure (2006) [7]–with emotional intolerance as one maintenance factor of the disorder—these authors recently further outlined their perspective on the aetiology of AN [19]. They described that vulnerabilities in social and emotional processing combined with a strong attention to detail and a reduced cognitive flexibility could increase the influence of social pressure at critical stages of development during adolescence and may thus allow the illness to develop.

To date, most inferences regarding the relation between emotional intolerance and AN-related symptoms have exclusively been drawn on the basis of cross-sectional data. In contrast, longitudinal data—in particular, when they are assessed on a daily basis—allow for conclusions about the temporal order of events and thus about the specific interactional dynamics between several processes. In other psychosomatic areas, longitudinal analysis by means of multivariate time series models has been conducted by an increasing number of researchers [20–23].

Additionally, in the majority of studies on emotion dysregulation, results were not differentiated based on levels of severity to assess whether relations differ for patients with a high symptom severity (HSS) vs. patients with a lower level of symptom severity (LSS). As such, these studies generally did not address patients with an extremely low body mass index (e.g., average BMI of 15.2 kg/m^2^ in [14]). Apart from a low BMI, serious social and psychological problems as well as a longer duration of illness before first inpatient treatment have been identified as additional risk factors of poor outcome in this disorder and can thus be considered as further characteristics of high severity in AN patients (e.g., [1;2]). Furthermore, there is increasing evidence that a long duration of illness, substance abuse, low weight, and poor psychosocial functioning enhance the mortality risk in AN [24].

Recently, Racine and Wildes (2015) and Haynos et al. (2015) published longitudinal findings on the relation between emotion dysregulation and AN symptoms [25;26]. Using ecological momentary assessment over a 2-week period, Haynos et al. aimed at evaluating whether restrictive eating enables AN outpatients to avoid contact with negative emotions. The findings of this initial investigation did not support an avoidance model of dietary restraint in AN patients. In particular, they observed stronger negative emotions on days characterised by high restriction. Racine and Wildes were interested in the temporal relations between emotion regulation difficulties and AN symptoms over the year following intensive treatment, hereby assessing questionnaires directly at discharge from treatment, as well as after 3, 6 and 12 months. The main finding indicates that emotion dysregulation predicted change in AN symptom severity over the year following discharge, but not vice versa. However, these studies only investigated patients with a relatively high BMI (Haynos et al.: mean BMI of 17.2 kg/m^2^; Racine & Wildes: mean BMI of 18.06 kg/m^2^ at baseline measurement [i.e., at discharge; with an average BMI at admission to treatment of 15.71 kg/m^2^]). In addition, they did not focus on the temporal dynamics during inpatient treatment.

### Objectives

The current study aims at investigating the dynamic interaction between emotional intolerance and AN symptoms over the course of inpatient treatment through a longitudinal design. Based on the previously described cross-sectional findings, we intended to find out whether significant temporal relations between emotional intolerance and AN-specific cognitions and behaviours such as dietary restraint and weight or eating concern would be found during the course of inpatient treatment. These temporal dynamics were hence compared for HSS (BMI < 15 kg/m^2^) vs. LSS patients.

As to the direction of these time-lagged relations, we expected that a higher degree of emotional intolerance would be followed by more intense AN symptoms in the majority of patients (and not vice versa). Additionally, we assumed that this would be particularly true for patients with a high level of symptom severity (HSS) and that less significant temporal relations between emotional intolerance and AN symptoms would be found in LSS patients.

## Materials and Methods

### Design

The study adopted a longitudinal, process-oriented observational design. It was approved by the medical ethics committee of the University Hospital Heidelberg (S404-2009). After a detailed description of the study to the participants, written informed consent was obtained. In total, 28 AN patients were enrolled. It is worth noting that a subgroup of these patients were subjects in a previous investigation of the characteristics of the cortisol awakening response in AN patients [27].

At the beginning of the study, the Structured Clinical Interview for DSM-IV (SCID) was conducted; all patients met the diagnostic criteria for AN. During the course of their inpatient stay, the patients answered questions daily before going to sleep on a handheld computer assessing a retrospective evaluation of the intensity of emotional intolerance and anorexic symptoms for each day. Thus, for each patient, we obtained time series of these self-assessments for the whole period of psychosomatic inpatient treatment.

### Sample

All participants were female AN inpatients meeting the DSM-IV criteria, over 18 years old with a BMI > 11 kg/m^2^ as well as sufficient physical and mental health to participate. Data collection took place during regular inpatient treatment on two wards. All eligible subjects were invited to participate during the first week of inpatient treatment. Out of 28 recruited patients, the data of only 16 patients could finally be included in longitudinal analysis. Four patients dropped out from study participation and two patients discontinued inpatient treatment against medical advice and thus could no longer take part in the study. In case of six patients, their diary data did not allow for the analysis of time series properties (e.g., when patients had restricted the majority of daily measurements to a single value). Subjects who passively or actively refused participation did not differ significantly from those included in data analysis with respect to age (*p* = 0.207) or BMI at admission (*p* = 0.801; unpaired *t*-test, two-tailed). The included 16 AN patients were admitted to two different wards. Nine patients were recruited from an integrated working psychosomatic and internal medicine ward (a group with high symptom severity, HSS). This ward—specialised in the treatment of AN patients with a very low BMI and usually a longer duration of illness—provides an intensive therapeutic schedule within a safe environment (e.g., supervised meal intake, supported weight management). The remaining seven patients were recruited from a psychotherapeutic ward (a group with low symptom severity, LSS) providing a specialised multimodal psychodynamic-oriented treatment for eating-disordered patients. Admission to the two different wards is indicated by the severity and chronicity of the illness. Patients with a BMI lower than 15 kg/m^2^, medically endangered patients, and patients with either a chronic course of the disorder or frequent relapses are preferentially admitted to the HSS ward. The characteristics of the analysed sample (N = 16) are described in Table 1.

**Table 1:** Characteristics of the analysed diary sample.

		HSS	LSS
		N = 9	N = 7
**female gender**	***N (%)***	100	100
**age (years)**	***mean (SD)***	23.2 (4.0)	25.1 (3.5)
	***range***	(19–30)	(23–32)
**ethnicity (Caucasian)**	***N (%)***	7 (77.8)	5 (71.4)
**educational level**: more than 9 years	***N (%)***	9 (100)	7 (100)
employment status*: currently working	***N (%)***	7 (77.8)	5 (71.4)
**marital status**: married or cohabiting	***N (%)***	0	2 (28.6)
**duration of illness (years)**	***mean (SD)***	5.4 (6.2)	3.4 (3.0)
	***range***	(1–17)	(1–10)
**subtype AN-R**	***N (%)***	8 (88.9)	6 (85.7)
**BMI at beginning of study (kg/m**^**2**^**)**	***mean (SD)***	13.5 (1.4)	16.2 (1.1)
	***range***	11.2–15.5	14.3–17.5
**BMI at end of study (kg/m**^**2**^**)**	***mean (SD)***	16.8 (1.8)	17.0 (0.6)
	***range***	14.4–19.0	16.4–17.7
**duration of participation (days)**	***mean (SD)***	123.1 (69.1)	68.9 (9.2)
	***range***	39–231	54–83
mental health comorbidities** **(at enrolment)**	***N (%)*:** major depression (current)	3 (30.3)	5 (71.4)
	***N (%)*:** minor depression (current)	1 (11.1)	1 (14.3)
	***N (%)*:** panic disorder (current)	2 (22.2)	2 (28.6)
	***N (%)*:** generalized anxiety (current)	1 (11.1)	-

AN-R: restrictive subtype

*currently working: having a job, working at home, studying or still at school

**diagnoses based on the Structured Clinical Interview for DSM-IV

Note: Out of N = 28 AN patients enrolled in the study, only N = 16 could be finally included in the time series analysis of the diary data (for reasons described in the text). Only the participants finally analysed are described in this table.

### Electronic Diary Measurements

After enrolment in the study, the patients received an electronic diary together with an individual training on how to use it. An alarm signal was used to remind the patients on a daily basis to complete the diaries over the course of their inpatient stay. In the diary, questions assessing the daily degree of emotional intolerance and AN-specific symptoms were implemented. The items were adapted from specific psychometric questionnaires. The inability to tolerate unpleasant emotions was assessed by means of one item taken from the Emotional Processing Scale (EPS; [28]). Concerning the assessment of the essential symptoms of AN, four items taken from the Eating Disorder Examination Questionnaire (EDE-Q; [29;30]) assessed restraint over eating, weight concern, fear of losing control over eating, and preoccupation with food, eating or calories. Additionally, one item integrating both nutritional and emotional aspects was implemented in the diaries (EPS; food impact on emotions). The particular items were chosen according to their psychometric properties in representing the variable of interest. Additionally, we took into consideration which items would be clinically most adequate for daily assessment. The diary items and details on the item selection process are provided in Table 2.

**Table 2:** Items implemented in the electronic diary.

	item	scale
emotional intolerance	“Today, I could not tolerate unpleasant emotions.”	EPS; subscale “avoidance” (*FL* = 0.81)
restraint over eating	“Today, I have been deliberately trying to limit the amount of food I eat to influence my shape or weight (whether or not I have succeeded).”	EDE-Q; subscale “restraint” (*r*_*it*_ = 0.82)
weight concern	“Today, my weight has influenced how I think about (judge) myself as a person.”	EDE-Q; subscale “weight concern” (*r*_*it*_ = 0.72)
fear of losing control	“Today, I had a definite fear of losing control over eating.”	EDE-Q; subscale “eating concern” (*r*_*it*_ = 0.75)
preoccupation with food	“Today, thinking about food, eating or calories made it very difficult to concentrate on things I am interested in (for example, working, following a conversation, or reading)?”	EDE-Q; subscale “eating concern” (*r*_*it*_ = 0.77)
food impact on emotions	“Today, the food eaten was responsible for my emotions.”	EPS; subscale “externalized” (*FL* = 0.47)

Items were chosen according to their psychometric properties in representing the variable of interest (*r*_*it*_: item-to-total correlations; *FL*: factor loadings). In particular, the focus items to assess emotional intolerance and dietary restraint display the highest factor loadings / item-to-total correlations among all subscale items. Additionally, we took into consideration which items would be clinically most adequate for daily assessment. *EPS (Emotional Processing Scale)*: As no validated German version of the EPS is available, two forward translations into German were completed by two translators who were German native speakers and fluent in English. After a reconciliation process, the German forward version on which both translators agreed was translated back into English by a native speaker of English and fluent in German (not involved in the previous steps in any way). This English backward version was then compared to the original English item. For both EPS items, the English back-translation and the original English items were identical. Therefore, the final German version of these items was used in our study. The factor loadings of the original English items are reported here [28]. *EDE-Q (Eating Disorders Examination Questionnaire)*: Items were taken from the validated German version of the EDE-Q [30], reporting on the item-to-total correlations of these items. The authors describe that the fourth EDE subscale “shape concern” cannot be sharply differentiated from the subscale “weight concern”. Given the necessity to design the diaries as short as possible in order to ensure daily compliance, we thus did not include a shape concern item.

Patients were asked to rate each item on a visual analogue scale (VAS) with bipolar labels. The marked points were converted by the computer program to a numeric scale, from 0 to 100, visible to the patient while completing the questionnaire. Handheld computers of type ARCHOS 5 Internet Tablet were used as electronic diaries. The “Octopus Mobile Survey Tool” was used as software for the mobile computers [31]. In addition to these items, patients were invited to note specific experiences or perceptions on each particular day in an open text field.

### Statistical Analysis

After examining linear time trends and same-day correlations, longitudinal diary data were analysed using a multivariate time series approach called vector autoregressive (VAR) modelling. This approach allows the investigation of temporal dynamics between several processes, and has been previously used in the domain of psychosomatic medicine (e.g., [20;32]). Its ability to analyse temporal relations between variables is frequently outlined as main advantage of this technique. In our study, statistical analysis was conducted according to the VAR procedure described in Rosmalen et al. (2012) [20], even more thoroughly in Stadnitski (2014) [33].

As preparatory steps, missing values in the time series were replaced by the regression algorithm implemented in SPSS Version 18. Furthermore, VAR modelling requires temporal stability of the participating processes. Thus, the augmented Dickey-Fuller (ADF) test was used to test for stationarity of the series [34;35]. It is worth remembering that the duration of inpatient treatment of AN patients usually differs remarkably according to their individual somatic and psychic status at admission. In particular, a very low BMI at admission requires a longer period of inpatient treatment. Therefore, clear-cut phases of inpatient therapy cannot be generally defined for all patients, but have to be determined individually for each of them. As restraint over eating is considered the most essential feature of AN, the dynamic interaction between emotional intolerance and this variable was of primary interest. Therefore, we first analysed whether these two variables displayed stationarity during the complete course of treatment. If yes, the complete period of treatment was used as time window to be analysed. If the remaining variables did not show to be stable during that time window, they could not be included in the VAR modelling process as we aimed at restricting time series analysis to one time window for all variables. If both focus variables did not fulfil the precondition of stationarity for the whole treatment duration, however, then stepwise visual inspection (starting from the first day of diary assessment) was applied in order to determine a distinct stable period in the focus variables.

Bivariate VAR analysis was then applied to all possible combinations of emotional intolerance with the AN symptom variables mentioned above. Hence, the following steps were conducted:

1. The best-suited number of lags required for the VAR model was determined by a stepwise use of lag length selection criteria. The criteria used were: Akaike Information Criterion (AIC), Final Prediction Error (FPE), Hannan-Quinn Information Criterion (HQIC), and the Bayesian Information Criterion (BIC).
2. Subsequently, diagnostic tests were used in order to check whether the final models were correctly specified. This includes testing the so-called eigenvalue stability condition to ensure the stability of the bivariate models. Additionally, the white noise assumption (no residual autocorrelation) was tested using Portmanteau tests and by inspection of the autocorrelation functions. In case a test indicated a violation of the model assumptions, the model was iteratively adjusted and re-estimated (starting with the most parsimonious number of lags) until all assumptions were met.
3. As a central step, the system’s dynamic behaviour was investigated by means of Granger causality testing, impulse response function (IRF) analysis and forecast error variance decomposition (FEVD). The Granger causality test indicates the directionality of the influence between two time series and thus the temporal order of events. A variable X is considered to Granger cause another variable Y if past values of X improve the prediction of Y (more than past values of Y alone can do) [36]. IRFs visualise the influence of a shock in one variable to the other variable thus displaying the dynamic impacts of changes in each of the variables over time. In case of additional contemporaneous (and not only time-lagged relations) orthogonalised IRFs (OIRFs) were calculated assuming that a specific ordering is chosen for the direction of this contemporaneous relationship. This choice has implications for the IRF itself as well as for the FEVD. Our decision for one particular ordering was guided by the direction of significant Granger causality. Additionally, accumulated IRFs were calculated in order to show the total impact over time (10 days) of a shock in the impact variable on the other variable. Finally, FEVD was calculated to estimate the amount of variance in a dependent variable that can be explained by a corresponding cause variable during a certain period of time.

The analyses were conducted using the freeware JMulti specifically designed for the analysis of multivariate time series models ([36]; available at http://www.jmulti.de).

## Results

**Fig 1:**
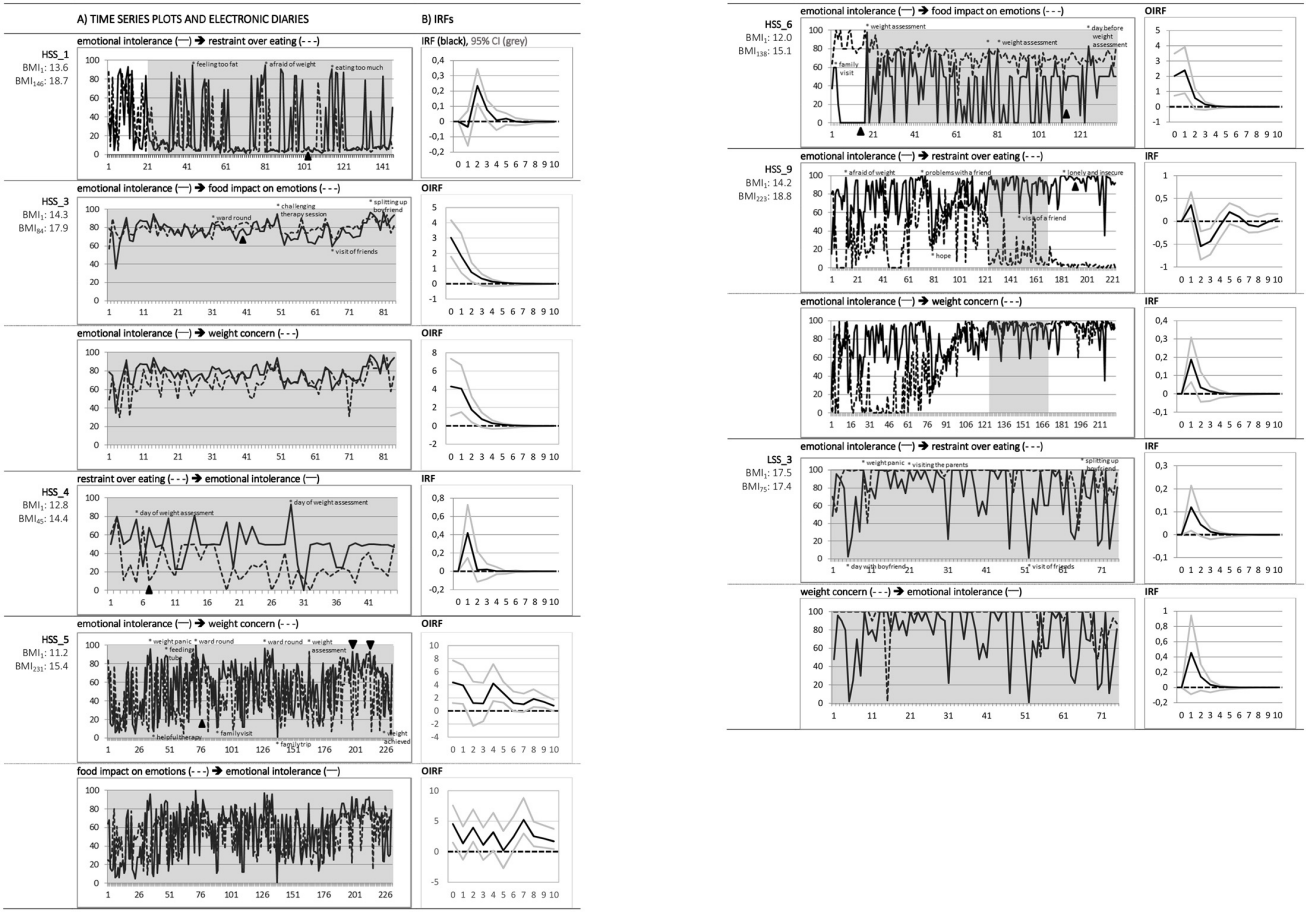
Plots of significant Granger causal relations combined with information from the electronic diaries as well as corresponding impulse response functions. Analysed time windows are shadowed in grey; family therapy sessions are marked with a black triangle; special notes from the diary are marked with *. Impulse response function (IRF) / orthogonalised IRF (OIRF): responses are considered significant if their error bands do not include 0. Confidence bounds are computed employing Hall bootstrap method. For a detailed description of IRF / OIRF see explanations in the text.

In the following, results regarding linear time trends, same-day correlations, and time-lagged relations between emotional intolerance and core features of AN during the course of inpatient treatment are described. Beforehand, plots of the time series (Fig 1) serve to illustrate these dynamics. For reasons of clarity and comprehensibility, only processes for which significant Granger causal relations were identified are graphically presented.

In the left column of Fig 1 (marked with A), time series plots are combined with information taken from the daily free text entries in the electronic diaries. BMI changes and family therapy sessions are listed as well. Inspecting the fluctuations of emotional intolerance (black line) reveals that extreme values in intolerable unpleasant emotions seem to be related with particular events. On a visual basis, it seems that weight- and food-related aspects (e.g., body perception of “being too fat” or notion of “eating too much”, stressful events of weight assessment on the ward) are associated with peaks in emotional intolerance. Consistently, peaks in emotional intolerance can be observed on days when challenging ward rounds and therapy sessions took place. In case of therapeutic interaction perceived as helpful by the patient, however, intolerable unpleasant emotions appear to decrease. Similarly, interaction with close others seems to be both combined with extremely high and extremely low values in emotional intolerance depending on the particular implication of that interaction for the patient (e.g., family therapy sessions). Below, findings on the interactional dynamics between emotional intolerance and AN-specific symptoms—as also reflected in Fig 1 –will be outlined.

### Linear time trends

Table 3 displays linear time trends significantly differing from zero in all the assessed variables during inpatient treatment for all the participating patients, stratified by symptom severity group.

**Table 3:** Linear time trends (standardised coefficients from time series regressions) in emotional intolerance and AN symptom variables during inpatient stay (in all patients).

		AN-specific behaviours and cognitions					
patient	emotional intolerance	food impact on emotions	restraint over eating	weight concern	fear of losing control	pre-occupation with food	T
HSS_1	-0.11	**-0.46**	**-0.42**	**-0.46**	**-0.31**	**-0.45**	146
HSS_2	**-0.50**	**-0.40**	**-0.28**	-0.08	**-0.43**	**-0.42**	91
HSS_3	0.14	**0.30**	**0.24**	**0.32**	0.02	0.20	84
HSS_4	-0.21	**-0.44**	**-0.33**	-0.15	-0.23	-0.26	45
HSS_5	**0.29**	**0.17**	-0.03	**0.14**	-0.13	**-0.27**	231
HSS_6	0.15	**-0.49**	**-0.53**	0.06	**-0.47**	**-0.57**	138
HSS_7	-**0.25**	**-0.42**	**0.23**	0.00	**-0.34**	-0.08	111
HSS_8	0.09	0.06	**0.67**	**-0.54**	-0.07	0.07	39
HSS_9	**0.44**	**-0.52**	**-0.39**	**0.76**	**-0.24**	**-0.44**	223
***HSS*:** *% sign*. *trends*	*44*.*4*	*88*.*9*	*88*.*9*	*55*.*6*	*55*.*6*	*55*.*6*	
*(neg*. *trends)*	*(neg*. *22*.*2)*	*(neg*. *66*.*7)*	*(neg*. *55*.*6)*	*(neg*. *11*.*1)*	*(neg*. *55*.*6)*	*(neg*. *55*.*6)*	
LSS_1	0.03	**0.47**	**0.35**	**0.29**	**0.49**	**0.54**	66
LSS_2	**-0.41**	**-0.43**	**-0.65**	**-0.29**	**-0.35**	**-0.45**	54
LSS_3	-0.18	-0.11	-0.13	-0.19	**-0**.**42**	**-0.36**	75
LSS_4	**-0.33**	-0.10	**-0.45**	-0.17	**-0.52**	**-0.27**	83
LSS_5	-0.03	0.09	-0.01	-0.22	**0.47**	**0.29**	66
LSS_6	**-0.66**	**-0.78**	**-0.38**	**-0.79**	**-0.83**	**-0.63**	73
LSS_7	0.18	0.03	**0.35**	0.08	-0.09	0.06	65
***LSS*:** *% sign*. *trends*	*42*.*9*	*42*.*9*	*71*.*4*	*42*.*9*	*85*.*7*	*85*.*7*	
*(neg*. *trends)*	*(neg*. *42*.*9)*	*(neg*. *28*.*6)*	*(neg*. *42*.*9)*	*(neg*. *28*.*6)*	*(neg*. *57*.*1)*	*(neg*. *57*.*1)*	

**HSS**: high symptom severity patients; **LSS**: low symptom severity patients. **T**: time series length. Note: coefficients significantly differing from zero (p < 0.05) are marked bold.

The majority of significant linear changes in emotional intolerance as well as AN symptoms can be observed in the HSS group, where each patient displays a significant trend in at least two of the variables. Here, the largest decrease of symptoms (negative trend) during treatment takes place in “food impact on emotions” (66.7%), directly followed by “restraint over eating”, “fear of losing control over eating” and “preoccupation with food” (all 55.6%).

In the LSS group, on the contrary, three out of seven patients do not show any significant decrease in any of the variables (vs. only one HSS patient with no significant decrease). Only four patients show changes in the majority of variables. Hereby, the percentage of significant negative trends is highest (57.1%) for the two “eating concern” items of the EDE-Q (“fear of losing control” and “preoccupation with food”; for item details see Table 2).

### Same-day correlations

The size of the calculated same-day Pearson correlations between emotional intolerance and AN symptom variables is up to medium for the HSS group and up to strong for the LSS group. Except for one patient (HSS_9), all of these correlations are positive. For both groups, emotional intolerance displays the three strongest contemporaneous correlations with weight concern (max. r_HSS_ = 0.54; max. r_LSS_ = 0.81), food impact on emotions (max. r_HSS_ = 0.49; max. r_LSS_ = 0.79), and preoccupation with food (max. r_HSS_ = 0.44; max. r_LSS_ = 0.80).

### Granger causal time-lagged relations

Table 4 summarises the main findings of this study on temporal relations between emotional intolerance and AN symptoms. The table lists the Granger causal relations between emotional intolerance and AN symptom variables that were identified by means of subsequential testing of all possible bivariate time series relations (statistical procedure described above). For the HSS group, the diary data of six out of nine patients (i.e., 66.7%) revealed significant results for the Granger causality test of emotional intolerance and AN symptoms. Note, that for three of these patients, not only one but two Granger causal relations could be identified (HSS_3, HSS_5, HSS_9). For the LSS group, however, significant time-lagged relations between emotional intolerance and AN symptoms could be found in only one out of seven patients (i.e., 14.3%). In the following, the results are therefore described for the HSS group. Concerning the direction of Granger causality, emotional intolerance is the impact variable in most of the cases. Thus, past values of emotional intolerance improve the prediction of restraint over eating (HSS_1, HSS_9), weight concern (HSS_3, HSS_5, HSS_9), and food impact on emotions (HSS_3, HSS_6) more than past values of these variables alone can do. Three cases show a Granger causal impact of restraint over eating (HSS_4), food impact (HSS_5), and weight concern (LSS_3) on emotional intolerance. Note that no Granger causal relations could be found between emotional intolerance and the eating concern subscales (fear of losing control, preoccupation with food).

**Table 4:** Lagged dependencies between emotional intolerance and AN symptom variables.

patients	analysed time window	type of dependency (direction of Granger causality)			VAR order	Granger causality test		VAR estimates (p < 0.05)		acc. IRF over 10 days (p < 0.05)	explained variance (10 days)	same-day correlation (p < 0.05)
						**F** (df_1_, df_2_)	**P**					
HSS_1	T21 –T146 (total 146)	*emotional intolerance*	→	restraint over eating	2	10.25 (2,238)	0.0001	+0.25	(t-2)	+0.3	15%	-
HSS_3	T1 –T84 (complete)	*emotional intolerance*	→	food impact on emotions	1	7.16 (1,160)	0.0082	+0.22	(t-1)	+6.17 (order 1)	29%	+0.49
		*emotional intolerance*	→	weight concern	1	8.92 (1,160)	0.0033	+0.45	(t-1)	+11.38 (order 1)	23%	+0.41
HSS_4	T1 –T45 (complete)	*emotional intolerance*	←	restraint over eating	1	9.40 (1,82)	0.0029	+0.42	(t-1)	+0.46	17%	-
HSS_5	T1 –T231 (complete)	*emotional intolerance*	→	weight concern	4	3.07 (4,436)	0.0165	+0.15	(t-1)	+23.83 (order 1)	11%	+0.26
		*emotional intolerance*	←	food impact on emotions	7	2.57 (7,418)	0.0133	+0.18	(t-2)	+28.39 (order 2)	16% (order 2)	+0.29
								+0.22	(t-7)			
HSS_6	T18 –T130 (total 138)	*emotional intolerance*	→	food impact on emotions	1	6.54 (1,218)	0.0112	+0.08	(t-1)	+5.17 (order 1)	12% (order 1)	+0.29
HSS_9	T125 –T170 (total 223)	*emotional intolerance*	→	restraint over eating	2	14.25 (2,78)	0.0000	+0.35	(t-1)	-0.6	37%	-
								-0.75	(t-2)			
		*emotional intolerance*	→	weight concern	1	10.28 (1,84)	0.0019	+0.19	(t-1)	+0.24	19%	-
LSS_3	T1 –T75 (complete)	*emotional intolerance*	→	restraint over eating	1	5.40 (1,142)	0.0216	+0.12	(t-1)	+0.18	9%	-
		*emotional intolerance*	←	weight concern	1	4.32 (1,142)	0.0395	+0.45	(t-1)	-	6%	-

**HSS**: high symptom severity patients; **LSS**: low symptom severity patients. **T**: measurements of the time series. **VAR**: vector autoregressive modelling. A significant **Granger causality test** implies that the first variable has causal impact on the second variable. The test statistics is F(df_1_,df_2_), where **df**_**1**_ is a number of tested restrictions (k) and **df**_**2**_ = 2T−4k−2 for bivariate VAR models with T as length of the time series and k as order of the VAR model. **(t-x)** specifies the lag of the significant VAR estimate. **Acc. IRF**: accumulated impulse response function (see details in text).

Whether these impacts are positive or negative can be seen from the signs of the estimates in the VAR models describing the mathematical factor (weight) by which past values of the impact variable are represented in current values of the other variable. All VAR estimates were tested against zero for all of the lags of each VAR model. Table 4 shows VAR coefficients significantly differing from zero. Apart from one particular exception, all of them display a positive sign. For the cases with emotional intolerance as impact variable, this implies that the more the patients could not tolerate unpleasant emotions on any one day during inpatient treatment, the more they would subsequently limit their food intake, be concerned about their weight or let their emotions be determined by the amount of food eaten. In a few patients, the temporal relation is reverse and the extent of restrictive eating, food impact of emotions and weight concern improves the prediction of emotional intolerance. The largest total impact over time (over 10 days) as described by the accumulated IRF of emotional intolerance on AN symptoms (food impact on emotions, weight concern) can be observed in HSS_3, HSS_5, and HSS_6.

By means of the FEVD, the percentage of variance that can be attributed to changes in the impact variable was determined. In restraint over eating, between 15% (HSS_1) and 37% (HSS_9) of variance can be explained by emotional intolerance, while restraint over eating itself explains 17% of variance in emotional intolerance (HSS_4). In weight concern, up to 23% of variance can be accounted for by changes in emotional intolerance, while up to 29% in food impact on emotions can be explained by emotional intolerance. Inversely, 16% of variance in emotional intolerance is explained by food impact (in HSS_5).

Additional significant contemporaneous correlations were found in HSS_3, HSS_5, and HSS_6 between emotional intolerance and weight concern as well as food impact on emotions (ranging between 0.26 and 0.49). Note, that these correlations were calculated for the specific time window under investigation.

As mentioned above, Fig 1 plots these significant Granger causal relations (A) and the IRFs (right column marked with B). The VAR analysed time window is shadowed in grey indicating that the found Granger causality reflects the dynamics of almost the complete inpatient stay for most patients. For two patients, the dynamics are differing for a period of adaptation (at the beginning of treatment). The fact that for one patient Granger causal interactions could be found only in a small time window during the second half of stay may be explained by the fact that she needed to undergo an urgent operation due to a periodontitis during the course of inpatient treatment (with consequences for her eating behaviour and weight gain). For the majority of cases, the IRF describes a significant impact of a change in emotional intolerance on eating restraint, weight concern, and the extent to which food intake was responsible for emotions. For a number of patients, contemporaneous relations are stronger than time-lagged associations (as indicated by orthogonalised IRFs).

## Discussion

To our knowledge, this is the first study to compare the temporal interaction of emotional intolerance and AN symptoms during psychosomatic inpatient treatment between patients with different levels of severity, hereby using a longitudinal design.

The main results of this study are the findings on *time-lagged temporal relations between emotional intolerance and anorexic symptoms*. In this concern, it is particularly interesting that the diaries of almost 70% of patients in the HSS group reveal significant Granger causal relations between emotional intolerance and AN symptoms (while this is true for only one LSS patient). Obviously, the inability to tolerate aversive emotions is strongly associated with AN-specific cognitions and behaviours, in particular dietary restraint and weight concern. For three of the HSS patients, however, multivariate time series analysis did not reveal significant temporal associations. A thorough screening of existing documentation on these patients does not allow for clear conclusions about common features that might explain this. Nevertheless, it is striking that one of these patients was discharged as no further progress could be observed. Another patient declined further inpatient treatment at an early stage.

Concerning the temporal order of events (direction of Granger causality), another important finding is that emotional intolerance is the impact variable in most of the cases and that this impact is positive (positive sign of VAR estimates). This is in line with the prediction of Schmidt and Treasure (2006, p. 357): “a decrease in avoidance of emotions will result in symptom improvement” [7]. FEVDs reveal that up to 37% of variance in restraint over eating, up to 29% in food impact on emotions and up to 23% in weight concern can be accounted for by changes in emotional intolerance. With regard to the persistency of the effects as described by the accumulated IRFs (over 10 days), particularly long-term influences of emotional intolerance can be observed on weight concern and food impact on emotions.

The results may be seen against the background of the treatment setting for HSS patients: from clinical experience, highly anorexic patients are extremely challenged on this psychosomatic ward as the setting continuously requires them to eat in order to stabilise their somatic status. It follows that they are confronted with manifold aversive feelings (e.g., anger, helplessness, despair) which they are not always able to deal with, preferring instead to avoid them. Hence, their inability to handle the intensity of these feelings is strongly associated with their anorexic behaviours and cognitions. This is in line with the recently described hypothesis that self-starvation with accompanying weight loss may serve as a dysfunctional behaviour to regulate aversive emotions in AN [37]. In contrast to this, emotional intolerance appears to be more loosely connected with AN symptoms in patients with less severe symptoms. Furthermore, in the treatment setting for LSS patients emotional intolerance is more directly addressed. The psychotherapeutic focus for LSS patients is predominantly on the perception and processing of emotions as well as on dissolving the close link between emotional experiences and responses by anorexic symptoms. Therefore, LSS patients may have learned to use alternative coping strategies.

Given the fact that both possible directions of Granger causality were tested systematically between emotional intolerance and the AN symptom variables (in each patient), one could infer that the findings support the notion that emotional intolerance in severely affected AN patients influences their psychopathology. However, against the complex background of AN aetiology and manifold existing models, this conclusion may be too simple. As outlined in the introduction, it has recently also been suggested that the functioning of the neuroendocrine system should be taken into consideration and that—following this aetiological concept—emotional symptoms could be considered as the consequence of the food deprivation that accompanies AN [17]. Despite being found in a minority of cases, it should not be overlooked that our analysis also revealed the opposite direction of Granger causality (AN symptoms → emotional intolerance). Also, in two patients both directions could be observed. Moreover, significant time-lagged relations between emotional intolerance and AN symptoms could be found in only one out of seven LSS patients. This could suggest that for LSS patients the temporal dynamics are different. Therefore, at this stage of longitudinal research it is not possible to clearly infer a pure mentalistic interpretation of the findings.

The findings on linear time trends and same-day correlations can be interpreted consistently. Based on a large number of repeated measurements, we were interested in how emotional intolerance and AN symptoms *change during inpatient treatment* and whether these linear time trends are significantly different from zero. In particular, we aimed at finding out, whether the same variables can be considered as “change factors” during the inpatient treatment of both HSS and LSS patients. Interestingly, in the HSS group each patient displays a linear change (mostly a decrease) in at least two of the variables during the course of inpatient treatment. This is different for LSS patients with almost half of them not showing any significant decrease in any of the variables. It also strikes the eye that most changes in the LSS group take place in the two “eating concern” variables while in the HSS group most changes can be observed in “restraint over eating” and “food impact on emotions”. The results on linear trends are in line with the finding that *contemporaneous correlations between emotional intolerance and AN symptoms* are significantly different from zero for most HSS patients, while this is only true for the minority of LSS patients. For both groups, the strongest same-day relations are found with weight concern, preoccupation with food and the impact of food on emotions.

Recently, idiographic approaches as used here are increasingly described in psychosomatic literature. Rosmalen et al. (2012) outline that between-subjects heterogeneity may be obscured by averaging results over a large sample while inter-individual differences are easier to detect using individual-based data analysis by means of time series models [20]. As demonstrated in the framework of this study, many psychosomatic variables show large intra-individual variability and thus require models to adequately capture these dynamics. Additionally, the used time series methods serve as tools to understand the dynamic time-lagged interaction between psychosocial and somatic factors, here emotional and food- or body-related aspects.

Our findings correspond to the results of Racine and Wildes (2015) who found that emotion dysregulation predicted change in AN symptom severity over the year after discharge from treatment [25]. In addition to the findings of Racine and Wildes, our results suggest that emotional intolerance is associated with changes in AN symptoms during treatment primarily in patients with a high level of symptom severity. The authors themselves discussed that their results might not generalise across different time frames and that ecological momentary assessment might be better suited to gain insights into immediate relationships between levels of emotion dysregulation and AN symptoms. Furthermore, they outlined the fact that they used composite measures of emotion dysregulation and AN, thus not reflecting differential temporal dynamics between specific emotional deficits or AN symptoms. Finally, they highlighted the restricted range of BMI in their AN sample.

To summarise, the present study has several *strengths*: statistical testing of temporal interaction, linear time trends and same-day correlations is exclusively possible in the framework of a multivariate time series design. Therefore, the study has used a person-specific paradigm to investigate the temporal relation between emotional intolerance and AN-specific symptoms using ambulatory assessments. Moreover, it displays high ecological validity as data were collected in the everyday-setting of the psychosomatic ward. In the framework of time series design, the present study is characterised by a particularly large number of repeated assessments over time (“longitudinal sample size”: up to 231 days). As data assessment for this kind of studies is demanding and time-consuming, hereby requiring an extraordinary compliance from the patients, the “cross-sectional sample size” (N = 16 analysed longitudinal datasets out of N = 28 originally enrolled patients) can be considered as remarkably high.

Our study has some *limitations*. In the same way as nomothetic results obtained at the group level do not necessarily have implications for individual patients, the generalisability of these idiographically gained findings to the population is limited.

Undoubtedly, it remains unclear, whether the intolerance of aversive emotions can be explained by difficulties in emotion regulation or rather by the variability of unpleasant emotions during the daily assessment period. Thus, future studies will need to further differentiate (e.g., by including further items in the diaries) whether the observed fluctuations are due to a rather general inability to regulate negative emotions or whether they can be understood as a reaction to specific events causing negative emotions on these days.

Concerning the self-assessment of emotional intolerance and AN-specific cognitions and behaviours using electronic diaries, it may be argued that results might have been deliberately manipulated by the patients. Considering the large number of observations and the variety of time trends observed in the process variables under consideration, this seems rather unlikely. However, implicit daily measures or external ratings might improve the assessment in future studies. Similarly, the variables under consideration were operationalised using a small amount of items in order to ensure the feasibility of daily participation. However, the items were thoroughly chosen by means of psychometric criteria such as item-to-total correlations. Moreover, daily assessment over a large period ensures sufficient reflection of the patients on each item. Still, future studies would be well served to capture more facets of emotion dysregulation on a daily basis.

The study revealed the inter-individual heterogeneity of treatment courses during psychosomatic inpatient stay of patients with AN, implying that presumably similar psychosocial and AN-specific aspects are relevant to the dynamics of this process but underlining that the way these aspects interact can be individually remarkably different. Nevertheless, the synopsis of these individual findings revealed that emotional intolerance in severely affected AN patients temporally precedes changes in their disorder-specific cognitions and behaviours. Therefore, the current study suggests that treatment of AN patients profits from focusing on the role of emotion regulation while at the same time adequate therapy of AN requires a thorough consideration of the individual case.

## References

[1] Arcelus J, Mitchell AJ, Wales J, Nielsen S: Mortality rates in patients with anorexia nervosa and other eating disorders: a meta-analysis of 36 studies. Arch Gen Psychiatry 2011;68:724–731. 10.1001/archgenpsychiatry.2011.74 21727255

[2] Zipfel S, Löwe B, Reas DL, Deter HC, Herzog W: Long-term prognosis in anorexia nervosa: lessons from a 21-year follow-up study. Lancet 2000;355:721–722. 10703806 10.1016/S0140-6736(99)05363-5

[3] Herzog W, Schellberg D, Deter HC: First recovery in anorexia nervosa patients in the long-term course: a discrete-time survival analysis. J Consult Clin Psychol 1997;65:169–177. 9103746 10.1037//0022-006x.65.1.169

[4] Klump KL, Buli CM, Kaye WH, Treasure J, Tyson E: Academy for eating disorders position paper: eating disorders are serious mental illnesses. Int J Eat Disord 2009;42:97–103. 10.1002/eat.20589 18951455

[5] Guarda AS, Pinto AM, Coughlin JW, Hussain S, Haug NA, Heinberg LJ: Perceived coercion and change in perceived need for admission in patients hospitalized for eating disorders. Am J Psychiatry 2007;164:108–114. 17202551 10.1176/ajp.2007.164.1.108

[6] Zandian M, Ioakimidis I, Bergh C, Södersten P: Cause and treatment of anorexia nervosa. Physiol Behav 2007;92:283–290. 17585973 10.1016/j.physbeh.2007.05.052

[7] Schmidt U, Treasure J: Anorexia nervosa: valued and visible. A cognitive-interpersonal maintenance model and its implications for research and practice. Br J Clin Psychol 2006;45:343–366. 17147101 10.1348/014466505x53902

[8] Haynos AF, Fruzzetti AE: Anorexia nervosa as a disorder of emotion dysregulation: evidence and treatment implications. Clin Psychol—Sci Pr 2011;18:183–202.

[9] Kyriacou O, Easter A, Tchanturia K: Comparing views of patients, parents, and clinicians on emotions in anorexia: a qualitative study. J Health Psychol 2009;14:843–854. 10.1177/1359105309340977 19786510

[10] Wildes JE, Ringham RM, Marcus MD: Emotion avoidance in patients with anorexia nervosa: initial test of a functional model. Int J Eat Disord 2010;43:398–404. 10.1002/eat.20730 19670226 PMC2882494

[11] Wildes JE, Marcus MD, Bright AC, Dapelo MM, Psychol MC: Emotion and eating disorder symptoms in patients with anorexia nervosa: an experimental study. Int J Eat Disord 2012;45:876–882. 10.1002/eat.22020 22473650 PMC3393827

[12] Kaye W: Neurobiology of anorexia and bulimia nervosa. Physiol Behav 22-4-2008;94:121–135. 10.1016/j.physbeh.2007.11.037 18164737 PMC2601682

[13] Brockmeyer T, Bents H, Holtforth MG, Pfeiffer N, Herzog W, Friederich HC: Specific emotion regulation impairments in major depression and anorexia nervosa. Psychiatry Res 30-12-2012;200:550–553. 10.1016/j.psychres.2012.07.009 22910477

[14] Brockmeyer T, Grosse HM, Bents H, Herzog W, Friederich HC: Lower body weight is associated with less negative emotions in sad autobiographical memories of patients with anorexia nervosa. Psychiatry Res 15-12-2013;210:548–552. 10.1016/j.psychres.2013.06.024 23850436

[15] Stroe-Kunold E, Wesche D, Friederich HC, Herzog W, Zastrow A, Wild B: Temporal relationships of emotional avoidance in a patient with anorexia nervosa—a time series analysis. Int J Psychiatry Med 2012;44:53–62. 23356093 10.2190/PM.44.1.d

[16] O’Hara CB, Campbell IC, Schmidt U: A reward-centred model of anorexia nervosa: A focused narrative review of the neurological and psychophyiosological literature. Neurosci Biobehav Rev 2015;52:131–152. 10.1016/j.neubiorev.2015.02.012 25735957

[17] Södersten P, Bergh C, Leon M, Zandian M: Dopamine and anorexia nervosa. Neurosci Biobehav Rev 2016;60:26–30. 10.1016/j.neubiorev.2015.11.003 26608248

[18] Riva G: Out of my real body: cognitive neuroscience meets eating disorders. Front Hum Neurosci 2014;8:236 10.3389/fnhum.2014.00236 24834042 PMC4018545

[19] Treasure J, Schmidt U: The cognitive-interpersonal maintenance model of anorexia nervosa revisited: a summary of the evidence for cognitive, socio-emotional and interpersonal predisposing and perpetuating factors. J Eat Disord 2013;1:13 10.1186/2050-2974-1-13 24999394 PMC4081714

[20] Rosmalen JGM, Wenting AMG, Roest AM, de Jonge P, Bos EH: Revealing causal heterogeneity using time series analysis of ambulatory assessments: application to the association between depression and physical activity after myocardial infarction. Psychosom Med 2012;74:377–386. 10.1097/PSY.0b013e3182545d47 22582335

[21] Stroe-Kunold E, Gruber A, Stadnytska T, Werner J, Brosig B: Cointegration methodology for psychological researchers: An introduction to the analysis of dynamic process systems. Br J Math Stat Psychol 2012;65:511–539. 10.1111/j.2044-8317.2011.02033.x 22070760

[22] Tschacher W, Ramseyer F: Modeling psychotherapy process by time-series panel analysis (TSPA). Psychother Res 2009;19:469–481. 10.1080/10503300802654496 19585371

[23] Wild B, Eichler M, Friederich HC, Hartmann M, Zipfel S, Herzog W: A graphical vector autoregressive modelling approach to the analysis of electronic diary data. BMC Med Res Methodol 2010;10:28 10.1186/1471-2288-10-28 20359333 PMC2869334

[24] Franko DL, Keshaviah A, Eddy KT, Krishna M, Davis MC, Keel PK, et al: A longitudinal investigation of mortality in anorexia nervosa and bulimia nervosa. Am J Psychiatry 2013;170:917–925. 10.1176/appi.ajp.2013.12070868 23771148 PMC4120076

[25] Racine SE, Wildes JE: Dynamic longitudinal relations between emotion regulation difficulties and anorexia nervosa symptoms over the year following intensive treatment. J Consult Clin Psychol 2015;83: 785–795. 10.1037/ccp0000011 25181027 PMC4345157

[26] Haynos AF, Crosby RD, Engel SG, Lavender JM, Wonderlich SA, Mitchell JE, et al Initial test of an emotional avoidance model of restriction in a norexia nervosa using ecological momentary assessment. J Psychiatr Res 2015;68:134–139. 10.1016/j.jpsychires.2015.06.016 26228412 PMC4522040

[27] Wild B, Wesche D, Schultz J-H, Stroe-Kunold E, Herzog W, Rudofsky G, et al: Trajectories of the cortisol awakening responses during weight gain in anorexia nervosa patients with severe and less severe symptoms. Int J Psychophysiol 2014;94:272–277. 10.1016/j.ijpsycho.2014.09.010 25286448

[28] Baker R, Thomas S, Thomas PW, Owens M: Development of an emotional processing scale. J Psychosom Res 2007;62:167–178. 17270575 10.1016/j.jpsychores.2006.09.005

[29] Fairburn CG, Beglin SJ: Assessment of eating disorders: interview or self-report questionnaire? Int J Eat Disord 1994;16:363–370. 7866415

[30] Hilbert A, Tuschen-Caffier B, Karwautz A, Niederhofer H, Munsch S: Eating Disorder Examination Questionnaire: Evaluation der deutschsprachigen Übersetzung. Diagnostica 2007;53:144–154.

[31] Bischof J, Kroll M. Software Projects. 2011. Available at http://octopus.softwareculture.bit

[32] Hoenders HJ, Bos EH, de Jong JT, de Jonge P: Temporal dynamics of symptom and treatment variables in a lifestyle-oriented approach to anxiety disorder: a single-subject time-series analysis. Psychother Psychosom 2012;81:253–255. 22678230 10.1159/000335928

[33] Stadnitski T: Multivariate time series analyses for psychological research: VAR, SVAR, VEC, SVEC models and cointegration as useful tools for understanding psychological processes. Hamburg, Kovac, 2014.

[34] Dickey DA, Fuller WA: Distributions of the estimators for autoregressive time series with a unit root. J Am Stat Assoc 1979;74:427–431.

[35] Stadnytska T: Deterministic or stochastic trend: decision on the basis of the Augmented Dickey-Fuller test. Methodol 2010;6:83–92.

[36] Lütkepohl H, Krätzig M: Applied time series econometrics. Cambridge, Cambridge University Press, 2004.

[37] Brockmeyer T, Holtforth MG, Bents H, Kammerer A, Herzog W, Friederich HC: Starvation and emotion regulation in anorexia nervosa. Compr Psychiatry 2012;53:496–501. 10.1016/j.comppsych.2011.09.003 22036318

